# 

**Published:** 1881-11

**Authors:** 


THE AMERICAN PUBLIC HEALTH ASSOCIATION will meet in Savannah, Ga., on the 29th of November. Dr. C. B. White, of New Orleans, is President of the Association, and Dr. Azel Ames, Jr., of Wakefield, Mass., is the Secretary.

THE CHAIR OF MATERIA MEDICA AND THERAPEUTICS, in the College of Physicians and Surgeons, Baltimore, made vacant by the sudden death of Prof. E. Lloyd Howard, has been filled by the election of Dr. Richard Gundry, of Spring Grove Asylum, to the vacancy.

### HYMENEAL.

HARBAN—HIGGINS—WALTER S. HARBAN, D. D. S., to Miss ISABELLA HIGGINS.—October 26th, 1881, Calvary Baptist Church, Washington, D. C., all of said city.

We regret our inability to be present on the nuptial occasion, but congratulate our former student on his departure from "single blessedness," and hope that he and his bonnie bride may ever continue in double happiness.

---

### NECROLOGICAL.

DR. CHAS. B. WHITE, Major and Surgeon, U. S. A., died August 10th, at Wilton, Conn., of recurrent adeno-sarcoma of the left axilla.

DR. J. G. HOLLAND died of Angina Pectoris in New York on Oct. 12th. Dr. Holland was a graduate in medicine, although most of his life had been devoted to literature.

Dr. J. M. Green, of the U. S. Marine Hospital Service, died recently at Key West, Fla., of yellow fever.

WILL *YOU* PLEASE SEND IN *YOUR* SUBSCRIPTION MONEY *NOW?*

Money is at Our Risk Only when sent by Post Office Money Order, Registered Letter, or Check on Banks in New York City, made payable to Independent Practitioner, 20 Courtlandt street, N. Y. City.

If you prefer to send check on your private bank, unless located in the city

